# School travel and children’s physical activity: a cross-sectional study examining the influence of distance

**DOI:** 10.1186/1471-2458-13-1166

**Published:** 2013-12-11

**Authors:** Guy Faulkner, Michelle Stone, Ron Buliung, Bonny Wong, Raktim Mitra

**Affiliations:** 1 Faculty of Kinesiology and Physical Education, University of Toronto, 55 Harbord Street, Toronto, Ontario M5S 2W6, Canada; 2 School of Health and Human Performance, Dalhousie University, 6230 South Street, PO Box 15000, Halifax, Nova Scotia B3H 4R2, Canada; 3 Department of Geography and Program in Planning, University of Toronto, 100 St.George St., Toronto, Ontario M5S 2W6, Canada; 4 School of Urban and Regional Planning, Ryerson University, Toronto, Ontario M5B 2K3, Canada

**Keywords:** Active travel, Built environment, Accelerometry, Children

## Abstract

**Background:**

Walking to school is associated with higher levels of physical activity. The purpose of this study was to examine the relationship between school travel mode and physical activity using a sampling frame that purposefully locates schools in varying neighbourhoods.

**Methods:**

Cross-sectional survey of 785 children (10.57 ± 0.7 years) in Toronto, Canada. Physical activity was measured by accelerometry and travel mode was self-reported by parents. Linear regression models accounting for school clustering effects examined the associations between mode choice, BMI, and physical activity and were estimated adjusting for age, types of neighbourhoods and travel distance to school.

**Results:**

Significant associations between walking to school and moderate activity during weekdays were found. Interactions between walking to school and travel distance to school were found only in boys with significant associations between walking to school and higher physical activity levels in those living within 1000–1600 meters from school. Boys walking to school and living in this range accumulated 7.6 more minutes of daily MVPA than boys who were driven.

**Conclusions:**

Walking to school can make a modest but significant contribution to overall physical activity. This contribution was modified by travel distance and not school neighbourhood socioeconomic status or the built environment.

## Background

The majority of Canadian and US children and youth do not accumulate the recommended amount of physical activity (PA) (i.e. 60 minutes of moderate-to-vigorous physical activity [MVPA] per day) needed for improved health [1,2]. The trip to school has been identified as one potential utilitarian source of physical activity. Active school travel (AST) has been the subject of targeted intervention designed to attenuate safety risks and increase the use of active modes both in the USA (e.g., Federal Safe Routes To School; http://safety.fhwa.dot.gov/saferoutes/funding) and Canada [3]. The potential health benefits of AST have also been acknowledged in both the public policy conversation (http://www.saferoutesinfo.org) and in published research. In the most recent systematic review, Larouche et al. [4] reported that AST was associated with higher physical activity levels in 22 of 28 studies using accelerometers, with mean differences in time spent in daily MVPA ranging from 0 to 45 minutes when compared to children who travel by car or public transport. With evidence that interventions can be effective in increasing active school travel [5], a focus on children’s school travel represents an opportunity to simultaneously address both public health and transport related environmental concerns [4].

In exploring the relationship between AST and physical activity levels, most studies tend to treat study populations as relatively homogenous [6]. For example, the location of the household in relationship to the school might be critical for a number of reasons. First, distance is consistently found to be negatively associated with AST [7]. While distance may be an AST barrier there may also be a dose–response relationship between AST and physical activity levels with increasing distance traveled by active means. Two UK studies demonstrate this effect [8,9]. Using parental self-reported distance, van Sluijs et al. [9] found modest differences in MVPA between walkers and children driven living within 0.5 miles from school. This increased to an additional 5.98 to 9.77 more minutes of MVPA for children living between 0.5 and 5 miles from school. Panter et al. [8] reported that the strength of association between walking to school and physical activity became stronger with increasing distance. Notably, there was some evidence for a compensatory effect in boys who walked to school; they were less active after school and at weekends.

Apart from distance, the built environment is assumed to both influence school travel decisions and physical activity. For children, evidence supports associations of reported physical activity with objective measures of walkability, traffic speed/volume, access/proximity to recreation facilities, land-use mix, and residential density [10]. Less consistent findings are reported for the relationship between school travel mode and measures of land use mix, residential density, and intersection density [7]. A household’s choices regarding opportunities for physical activity, the safety of engaging in physical activity, and school travel mode options are also affected by socioeconomic status (SES). Accordingly, the relationship between school travel mode and physical activity levels may be influenced at a neighbourhood level in terms of differences in built environment features and socioeconomic status. To the best of our knowledge, no study has examined the relationship between AST and objective measures of physical activity using a sampling strategy designed to capture sufficient representation of variation in school neighbourhood characteristics.

In this study, we examined the relationship between school travel mode, distance, and objectively measured physical activity and body mass index (BMI) using a sampling frame that purposefully located schools in varying neighbourhoods in the city of Toronto, Canada. The City of Toronto includes both central city and inner suburban neighbourhoods. Neighbourhoods within the central city can be characterized as being older traditional neighbourhoods, largely constructed prior to World War II [11]. Toronto’s inner suburban neighbourhoods were largely developed following World War II, and include Canada’s earliest examples of suburban residential neighbourhood designs that have come to dominate much of the North American suburban landscape. More recently, there has been a concentrated effort to intensify development along Toronto’s major arterials, and there has been a trend toward the redevelopment of employment lands as high-density condominium neighbourhoods. Toronto’s inner suburbs also include the high-density tower apartment neighbourhoods. With these exceptions in mind, era of development can serve as a proxy for neighbourhood type. Despite localized changes in density and land use, Toronto’s road network has not undergone a major transformation in the face of new development. Central city streets often follow a grid iron pattern, while inner suburban streets typically possess the hallmark qualities of modern design, hierarchically ordered curvilinear streets serving often segregated land uses [11,12]. Socioeconomic status also varies quite markedly both within and across central city and inner suburban neighbourhoods. Sampling across neighourhood era of development and neighbourhood socioeconomic status allowed an examination of how broader social and environmental determinants of behavior might modify the relationship between school travel mode and physical activity levels among elementary school children.

## Methods

### Experimental design

From January 2010 to June 2011, all elementary/intermediate schools within the Toronto District School Board (TDSB) with Grade 5 and 6 students (n = 469) received an invitation to participate in the study. A pool of interested schools (n = 54 responded, 40 of which were interested; response rate = 11.5%) was generated and 16 schools were selected based on the built environment and socioeconomic status (SES). First, the period of neighbourhood development and typical street layout near a school location was considered. School neighbourhoods in which > 50% of residential units were developed prior to 1946 (computed at the scale of census dissemination area, DA) and where the streets typically followed gridded layout were classified as old neighbourhoods. Street layout was confirmed by visual inspection. In contrast, neighbourhoods that were largely developed after 1946 and had curvilinear streets with a clear hierarchy of road systems were identified as old neighbourhoods. DAs are the smallest geographical units that census data by Statistics Canada are available. The year 1946 was selected as a proxy for pre and post-World War II neighbourhoods.

Second, neighbourhood SES (low SES and high SES) was modeled using median household income. For each TDSB school, the median household income within an 800 m straight line buffer was computed by taking the median of the household incomes of DAs that fell in the buffer. Schools with the lower 50 percentile values were identified as Low SES. Four schools were selected from neighbourhood strata (old, low SES neighbourhood; old, high SES neighbourhood; new, low SES neighbourhood; and new, high SES neighbourhood) developed by intersecting period of neighbourhood development and neighbourhood-level SES. Ethics approval was granted by the University of Toronto Research Ethics Board. Consent was granted by the participating school board, individual schools, parents and students.

A total of 1027 from 1704 eligible parents/guardians at the 16 schools gave consent for their children to participate (boys, *n* = 478; girls, *n* = 549). Height and weight measurements were taken and accelerometer-measured physical activity data collected on a total of 1001 children. Of these children, 85.5% had at least 3 weekdays and 1 weekend day of valid data (*n* = 856; boys = 389, girls = 467). For inclusion in data analysis, each child required a minimum of 10 hours of accelerometer wear time for at least three weekdays and one weekend day. A string of thirty minutes of consecutive zeros was used to classify non-wear time/account for time spent in sleep; these periods (most of which occurred during sleep) were removed from analyses. Among 856 children with valid data, 785 children [boys = 357(45.5%) and girls = 428(54.5%)] who walked or were driven were included in the analyses for a final response rate of 46% (i.e., 1704/785 × 100). Mean age was 10.57 years (SD = 0.7 years). Children with other travel modes (n = 54; public transit or school bus) and cycling (n = 17) were excluded. There were no significant differences between students with valid versus invalid accelerometer data in terms of age, gender, usual school travel mode, and BMI.

### Measures

#### Physical activity

Children’s physical activity behaviour was objectively measured using accelerometers (ActiGraph GT1M; Pensacola, FL) for seven consecutive days (week). Time spent at different levels of intensity was classified according to published thresholds in children [13]. Physical activity measures of interest included total counts (counts/day), mean counts (counts/min), time spent sedentary (min) and time spent in moderate, vigorous and moderate-to-vigorous intensity activity (min) across the week and on weekdays and the weekend (note: see [14] for further details of the accelerometry data collection and analysis protocol).

#### Anthropometry

Weight and height were measured while children were in light clothing. Body Mass Index (BMI) was calculated by dividing weight (kg) by squared height (m^2^).

#### School travel

Parents completed a survey assessing household demographics (e.g., age and sex of child). To assess school travel mode, one item asked ‘in the morning, how does your child usually get to school?’ Options included: walk, ride a bicycle, school bus, public transit (subway, streetcar or city bus), or driven in a vehicle (car, truck, or van). A similar item used in the US National Center for Safe Routes to School (SRTS) program has demonstrated high parent-student convergent validity of school travel mode and parental test-retest reliability [15]. Only walkers and children who were driven in a vehicle were included in these analyses.

### Modifiers and covariates

Neighbourhood types were determined by the period of neighbourhood development and SES. The resulting four neighbourhood classifications were: 1) old low SES; 2) old high SES; 3) new low SES and 4) new high SES. To assess distance to school from home, children were asked to draw their route to school on an image map (i.e., an orthorectified image with street centerlines and labels added) of their neighbourhood. These school routes were then digitalized and measured, using the ArcGIS Geographic Information Systems (GIS) software. Neighbourhood type and distance to school were treated as potential modifiers. Age was assessed in the parental questionnaire and served as a covariate.

### Analyses

785 children with complete parental survey and accelerometry data, and data on their route to school, were included in the analyses. Linear regression models with robust standard errors accounting for school clustering effects were used (Stata 11). The associations between mode choice, BMI, physical activity levels were initially estimated adjusting for age, neighbourhood type and travel distance to school. Interaction terms of i) neighbourhood type and ii) travel distance to school by mode choice were added to a main effects model separately. The modifying effects of neighbourhood type and distance on the associations between mode choice, BMI, physical activity levels and meeting current physical activity guidelines were estimated using post-estimation linear combinations of regression coefficients [16]. The main analyses were stratified by gender due to the expectation of gender differences in activity patterns and correlates [17].

## Results

Overall, most children walked to school (72%). However, this varied by neighbourhood, with car travel increasing when moving from older, lower SES neighbourhoods (16%), to older, higher SES neighbourhoods (19%), to newer, lower SES neighbourhoods (28%) and newer, higher SES neighbourhoods (52%). Mean distance from home to school in these neighbourhoods was 1053.01, 887.30, 683.40, and 1355.98 meters respectively. Boys were more likely to engage in activity of ≥ moderate intensity (p < 0.05; see Table 1). In both boys and girls, a significant association between walking to school and the accumulation of moderate intensity activity during weekdays was found (β_ModerateMale_ = 3.6, p < 0.001; β_ModerateFemale_ = 3.9, p < 0.05) (Table 2). Boys but not girls who walked to school were also more likely to engage in greater MVPA (β = 4.1; p < 0.01). No associations between walking to school and BMI were found. There were no statistically significant differences in weekend physical activity characteristics between walkers and those driven during the week.

**Table 1:** Descriptive characteristics of the sample

	***All (n = 785)***		***Boys (n = 357)***		***Girls (n = 428)***	
	***Mean***	***SD***	***Mean***	***SD***	***Mean***	***SD***
BMI*	18.966	3.551	19.398	3.889	18.605	3.203
Total counts (counts/day)						
Week*	413457.7	123847.3	465198.2	122745.3	370300.2	107217.1
Weekdays*	438802.4	133234.2	497569.6	137395.1	389783.9	107509.3
Weekend*	347997.4	158595.7	382917.4	158595.7	382917.4	158585.8
Mean counts (counts/min)						
Week*	429.7	147.8	477.6	152.9	389.6	130.8
Weekdays*	449.9	157.0	504.9	167.7	404.0	130.9
Weekend*	377.1	181.4	407.2	180.0	352.0	178.9
Moderate intensity activity (min)						
Week*	22.4	10.0	27.5	10.2	18.2	7.5
Weekdays*	24.4	11.0	30.1	11.3	19.5	8.1
Weekend*	17.5	11.0	20.9	12.1	14.7	9.2
Vigorous intensity activity (min)						
Week*	5.9	4.2	7.1	4.4	4.8	3.6
Weekdays*	6.5	4.6	8.0	5.2	5.3	3.7
Weekend*	4.3	4.6	4.9	4.4	3.7	4.6
MVPA (min)						
Week*	29.3	13.9	35.6	14. 2	24.1	11.2
Weekdays*	31.9	15.3	39.2	16.0	25.9	11.7
Weekend*	22.6	15.3	25.5	16.1	19.3	13.8
Travel distance to school (%)						
0-500 m	34.4		37.8		31.5	
501-1000 m	36.8		38.9		35.0	
1001-1600 m	17.5		16.0		18.7	
>1600 m	11.3		7.3		14.7	
School location (%)						
Old, low	22.0		23.8		20.6	
Old, high	26.6		24.6		28.3	
New, low	29.2		29.4		29.0	
New, high	22.2		22.1		22.2	

Footnote: Old, low: old neighbourhood, low SES; Old, high: old neighbourhood, high SES; New, low: new neighbourhood, low SES; New, high: new neighbourhood, high SES; *Significant gender difference p < 0.05.

**Table 2:** The associations between active school travel, BMI, and physical activity

	***All (n = 785)***		***Boys (n = 357)***		***Girls (n = 428)***	
	***β***	***95% CI***	***β***	***95% CI***	***β***	***95% CI***
BMI	0.1109	-0.584,0.806	0.129	-0.659,0.916	0.003	-1.305,1.311
Total counts (counts/day)						
Week	30945.5**	9670.8,52220.3	33590.0**	8368.2,58811.7	28479.3	-9498.9,66457.4
Weekdays	37064.8*	14968.4,59161.1	42212.8**	17847.8,66577.8	30978.6	-11562.1,73519.2
Weekend	17470.2	-12502.8,47443.3	14392.9	-23223.9,52009.8	22158.5	-27217.5,71534.4
Mean counts (counts/min)						
Week	24.3	-2.8,51.5	38.8*	8.6,69.0	3.5	-48.9,55.9
Weekdays	30.7*	1.6,59.8	48.9**	18.9,78.9	4.7	-54.9,64.4
Weekend	11.6	-21.2,44.4	17.1	-24.6,58.9	2.2	-51.2,55.7
Moderate intensity activity (min)						
Week	2.8***	1.2,4.4	2.8**	0.9,4.6	2.9	-0.03,6.0
Weekdays	3.6***	1.8,5.4	3.6***	1.7,5.5	3.9*	0.5,7.4
Weekend	0.9	-1.1,2.9	0.8	-1.5,3.18	0.9	-2.7,4.5
Vigorous intensity activity (min)						
Week	0.1	-0.6,0.8	0.1	-0.8,0.8	0.3	-0.8,7.6
Weekdays	0.3	-0.5,1.1	0.2	-0.6, 1.0	0.6	-0.3,9.6
Weekend	-0.2	-1.1,0.7	-0.1	-1.3,1.1	-0.4	-4.3,5.2
MVPA (min)						
Week	3.1**	0.8,5.3	3.1*	0.5,5.6	3.3	-1.0,1.7
Weekdays	4.1**	1.6,6.6	4.1**	1.5,6.7	4.6	-1.1,2.3
Weekend	0.7	-2.0,3.5	0.9	-2.5,4.3	0.5	-1.7,0.9

Footnote: ***p < 0.001; **p < 0.01; *p < 0.05. β, Exponentiated beta coefficients; CI, confidence interval; MVPA, moderate-to-vigorous physical activity. The models were adjusted for age, neighbourhood type, distance to school, (and sex in models for whole sample).

### Modifying effects: travel distance to school and neighbourhood

**Figure 1:**
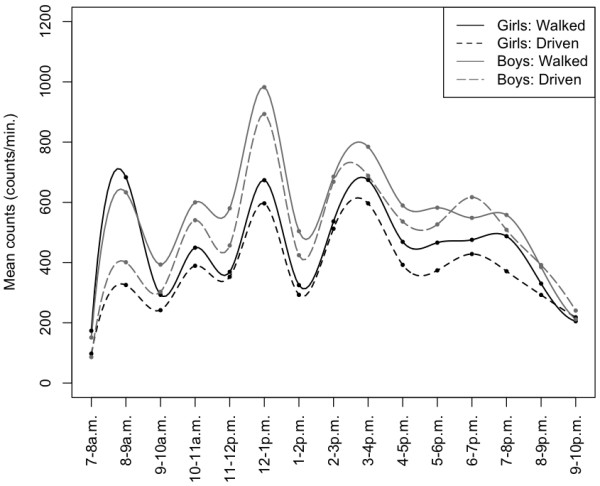
Mean accelerometry counts/minute for children living 1001-1600 metres from school.

There were no significant interactions between travel mode and neighbourhood type in boys or girls in terms of any physical activity measure. Interaction between walking to school and travel distance (as modifier) to school was detected, with strongest associations between walking to school and higher physical activity level in those living within 1000–1600 meters from school; this effect was strongest for boys (Table 3). For example, male walkers who lived within 1000–1600 meters from school engaged in greater total physical activity (β_Counts/minMale_ = 79.6, p = 0.05), more moderate (β_ModerateMale_ = 6.1, p = 0.02) and moderate-to-vigorous intensity activity (β_MVPAMale_ = 7.6, p = 0.03) during weekdays than boys driven. Figure 1 presents the average weekday physical activity pattern for the two travel groups in this distance category. Girls who walked to school were significantly more active than girls driven before school (7–8 a.m. and 8–9 a.m.) and also after school (3–4 p.m. and 5–6 p.m.) (p < .02). Boys who walked to school were significantly more active than boys driven only before school (7–8 a.m. and 8–9 a.m.; p < .02).

**Table 3:** The associations between active school travel, BMI, and physical activity levels modified by distance to school (variables with significant associations shown)

	***All (n = 785)***		***Boys (n = 428)***	
	***β***	***95% CI***	***β***	***95% CI***
Total counts (counts/day)				
Week	P = 0.04			
0–500 m	-50213.1	-120094.4,19668.2		
501–1000 m	20659.1	-10053.1,51371.4		
1001–1600 m	63773.1***	28633.4,98912.7		
>1600 m	41555.7	-13311.8,96423.1		
Weekdays	P = 0.03			
0–500 m	-17638.7	-78480.8,43203.4		
501–1000 m	22132.8	-12344.7,56610.3		
1001–1600 m	73435.5***	37674.3,109196.7		
>1600 m	40286.6	-15650.9,96224.0		
Mean counts (counts/min)				
Week	P < 0.001		P = 0.05	
0–500 m	-73.1*	-145.7,–0.4	-59.6	-153.1,34.0
501–1000 m	7.0	-35.9,50.0	27.3	-18.5,73.0
1001–1600 m	67.2**	23.7,110.7	79.6***	34.0,125.1
>1600 m	44.1	-18.8,107.1	59.0	-12.4,130.5
Weekdays	P = 0.02			
0–500 m	-46.3	-120.2,27.7		
501–1000 m	7.2	-40.6,55.00		
1001–1600 m	78.3***	34.1,122.5		
>1600 m	48.0	-18.6,114.5		
Moderate intensity activity (counts/min)				
Week	P = 0.03		P = 0.02	
0–500 m	-3.8	-9.2,1.65	-5.2	-11.5,1.1
501–1000 m	2.1	-0.1,4.2	2.2	-0.1,4.5
1001–1600 m	5.0***	2.2,7.9	5.6***	2.3,9.0
>1600 m	4.1*	0.0,8.3	4.3*	0.1,8.5
Weekdays			P = 0.02	
0–500 m			-1.6	-5.2,2.1
501–1000 m			2.4	-0.3,5.2
1001–1600 m			6.1**	2.3,10.0
>1600 m			5.6*	1.2,10.0
MVPA (min)				
Week	P = 0.04		P = 0.03	
0–500 m	-5.8	-13.0,1.4	-7.7	-16.0,0.6
501–1000 m	2.2	-0.9,5.4	2.7	-0.8,6.2
1001–1600 m	5.9**	2.0,9.9	6.8**	2.4,11.2
>1600 m	4.6	-1.3,10.6	4.3	-1.7,10.3
Weekdays			P = 0.03	
0–500 m			-3.4	-8.9,2.1
501–1000 m			3.0	-0.9,6.9
1001–1600 m			7.6**	2.5,12.7
>1600 m			5.3	-0.8,11.5

Footnote: ***p < 0.001; **p < 0.01; *p < 0.05; non-significant interactions are not reported. β, Exponentiated beta coefficients; CI, confidence interval; MVPA, moderate-to-vigorous physical activity. The models were adjusted for age, neighbourhood type (and sex in models for whole sample) with distance to school as a modifier.

## Discussion

In this sample active school transport made a significant contribution to overall levels of physical activity. No significant differences in this or the other physical activity outcomes were observed between travel groups on weekends. This suggests that differences during the week cannot simply be explained by natural differences in activity levels of different groups of children. Clearly, there is now consistent evidence that the school trip provides a significant source of physical activity for children across different countries, cultures, and school systems [4]. A recent study of 9–10 year old children in the UK reported 7 minutes more MVPA in children who walked/cycled to school compared with those who travelled by car [18]. While the overall contribution of AST to daily MVPA in the current study (i.e., approximately 4 minutes) is modest, the results here are influenced by the application of a more stringent threshold (≥4 METs) to classify MVPA. However, recent evidence suggests that this threshold may be more appropriate for describing ≥ moderate intensity activity in children [19-21].

The novel contribution of the study was exploring how the relationship between physical activity and travel mode may vary on the basis of school neighbourhood location – variation in neighbourhood socioeconomic status and era of development may modify the relationship given what we know about how these factors can influence both physical activity behaviour and travel decisions in general. Our findings suggested that the type of neighbourhood that schools were located in (for example, those characterized by more gridded or looping street architecture) was not important in terms of the relationship between travel mode and physical activity levels. Rather, and in a somewhat confirmatory sense, irrespective of neighbourhood type, living within a walkable distance from school was most significant. The strongest distance effect was observed for boys living between 1000 and 1600 metres from school. It is worth noting that in the study context bus services are typically offered for elementary school student at a 1.6 km cutoff, and at this point clear declines in walking are observed.

Our findings then reinforce the overwhelming importance of school proximity for both increasing the likelihood of walking, and in providing a significant source of physical activity for children. In terms of policy implications, school siting decisions are consequently integral to any discussion of promoting active school travel, while other strategies to increase the number of households located between 1 and 1.6 kms could also be considered such as reconsidering the size of school catchment areas. Perhaps more critical than the issue of school siting is the conversation about school closure. Discussion about the economically rational delivery of public education often turns to the issue of school closure [22]. Loss of neighbourhood schools could have the unintended consequence of increasing average distance traveled. Evidence from this study suggests that such neo-liberal policies could work against the travel mode and child health goals and benefits of safe routes to school initiatives. Extending the cutoff distance for school bus eligibility even modestly (e.g., from 1.6 to 2 km in the Toronto District School Board) might also be a policy approach to increase walking and daily MVPA among children. Of course, low traffic exposure and high connectivity [23] may be necessary preconditions for facilitating greater walking, yet these may be less influential overall than the distance between home and school – particularly in terms of the accumulation of MVPA.

Most studies report that children who walk to school are more active in general [4]. However, at least four studies using accelerometers have found that differences in PA were only significant among boys [15,24-26]. Similarly, we found that associations between travel mode and physical activity were stronger in boys. Reasons for this can only be speculated. Figure 1 demonstrates a gender difference in accelerometer mean counts among boys and girls throughout the day (living within 1 and 1.6 km from school). However, exceptions to this are when girls who walk are compared to boys driven before and after school. Girls who walk were significantly more active than both boys and girls who were driven before school, and as active as boys driven during the immediate after school period. That is, there is gender equalization in physical activity during these specific periods of the day, which might be lost when considering the total daily volume of physical activity. Given overwhelming evidence of gender-discrepancies in physical activity amongst Canadian children (with girls less active and less likely to achieve PA guidelines [27]), this realization that the trip to school may be a particularly valuable opportunity for MVPA for girls is quite promising. Fewer girls engage in AST [28,29] and parental perceptions regarding school travel are different for girls than they are for boys [30]. Future AST intervention work should explore the potential for gender-specific tailoring that addresses the different barriers girls may face to AST.

### Strengths and limitations

The strengths of the study included its relatively large sample, purposive sampling frame, an objective measure of physical activity, and accurate estimates of distance to school using GIS to digitize actual routes taken to school as reported by parents and children. Limitations included its cross-sectional design, the final response rate and sample representativeness. Although consistent with other active-consent studies with Canadian elementary school students [31], our sample was less than 50% of the eligible population after data screening. One related factor is also the possibility of response bias. The prevalence of walking to school in our sample (72%) was higher than that reported in population-based cross-sectional travel surveys conducted in the Greater Toronto region (48.1%) [28]. Driving households may have been reluctant to participate in the study if they interpreted it as pro-walking. Attempts were made to emphasize that the study was about the built environment rather than travel mode per se. Finally, since Toronto’s public schools maintain small catchment areas, this research assumed that the socioeconomic and built environment near school and home locations would generally be similar (1.6 km between school and home). This may not be the case and caution is required in ruling out the influence of neighbourhood design on the relationship between travel mode and physical activity. The present study did not examine the influence of micro-level community design and land-use characteristics (e.g., connectivity; access/proximity to recreation facilities, residential density, land use mix) and this is a focus for future work.

## Conclusions

This study demonstrates that walking to school is associated with higher levels of MVPA in comparison to children who are driven to school. There is a dose–response relationship with differences in MVPA between walking and driven children being greater when children lived within 1 and 1.6 km from school. This relationship was primarily evident for boys. Intervention studies are needed that focus on increasing walking among households where children are typically driven and that are located within 1.6 km of school. Our study also demonstrates gender convergence in physical activity during the morning and after-school periods when comparing girls who walk to school with boys who are driven. Since girls are less likely to use AST than boys, there is a definite need to tailor interventions to address the different barriers girls and boys may face in walking to school. Finally, broader policy approaches are required to ensure school proximity is within such a distance for the majority of households at the elementary school level.

## Competing interests

We declare that we have no competing interests.

## Authors’ contributions

GF and BW analyzed and interpreted the data and developed the first draft of the manuscript. GF and RB contributed to the conception and the design of the study. MS and RM contributed to the study development and interpretation of the data. All authors provided critical feedback during manuscript development. Each author has read and approved the final manuscript.

## Authors’ information

GF is a Professor in the Faculty of Kinesiology and Physical Education, University of Toronto. MS is an Assistant Professor in the School of Health and Human Performance, Dalhousie University. RB is an Associate Professor in the Department of Geography and Program in Planning, University of Toronto. BW is a graduate student in the Faculty of Kinesiology and Physical Education, University of Toronto. RM is an Assistant Professor in the School of Urban and Regional Planning, Ryerson University.

## Pre-publication history

The pre-publication history for this paper can be accessed here:

http://www.biomedcentral.com/1471-2458/13/1166/prepub
